# Late miscarriage and stillbirth in asymptomatic and symptomatic hospitalised pregnant women in Belgium during the first and second waves of COVID-19: a prospective nationwide population-based study

**DOI:** 10.1186/s12884-023-05624-3

**Published:** 2023-05-16

**Authors:** An Vercoutere, Mbiton Joel Zina, Karolien Benoit, Elena Costa, Sara Derisbourg, Michel Boulvain, Kristien Roelens, Griet Vandenberghe, Caroline Daelemans, on behalf of the B. OSS collaborating group, J. Ackermans, D. Anton, M. Bafort, A. Batter, Julie Belhomme, A. Beliard, B. Bollen, V. Boon, Jan Bosteels, V. Bracke, Gilles Ceysens, F. Chaban, Frédéric Chantraine, E. Christiaensen, L. Clabout, P. Cryns, M.-C. Dallequin, Bart De Keersmaecker, J. De Keyser, A. De Knif, Petra Scheir, Jeff De Loose, A. De Vits, Toenga De Vos, B. Debecker, C. Delforge, J. Deloor, V. Depauw, A. Depierreux, K. Devolder, L. Claes, S. Dirx, C. Eerdekens, Patrick Emonts, E. Goenen, P. Grandjean, S. Hollemaert, Sylvie Houben, E. Jankelevitch, G. Janssen, J. Quintelier, Yasmine Kacem, C. Klay, A. Laurent, J.-F. Legrève, A. Lestrade, C. Lietaer, A. Loccufier, Hilde Logghe, F. Loumaye, V. Mariman, N. Minten, D. Mortier, K. Mulders, G. Palgen, Thomas Pezin, K. Polisiou, Catherine Riera, M. Romain, Benoit Rombaut, M. Ruymbeke, K. Scharpé, C. Schockaert, A. Segers, Elvira Serkei, Patricia Steenhaut, An Steylemans, B. Thaler, W. Van Dalen, E. Van De Poel, E. Van Deynse, R. Van Dijck, Caroline Van Holsbeke, L. Van Hoorick, G. Van Olmen, P. Vanballaer, Kristel Vancalsteren, S. Vandeginste, S. Vandepitte, K. Verbeken, A. Vereecke, M. Verheecke, L. Watkins-Masters, V. Wijckmans, K. Wuyts

**Affiliations:** 1 Department of Obstetrics and Gynaecology, Hôpital Universitaire de Bruxelles (H.U.B), CUB Hôpital Erasme, Université Libre de Bruxelles (ULB), Anderlecht, Belgium; 2 Outbreak Support Team Carolo, Hainaut, Belgium; 3 Belgian Obstetrical Surveillance System, Ghent University Hospital, Ghent, Belgium; 4 Department of Obstetrics and Gynaecology, Ghent University Hospital, Ghent, Belgium; 5 Department of Human Structure and Repair, Ghent University, Ghent, Belgium; 6 Department of Woman, Child and Adolescent Medicine, Geneva University Hospitals, Geneva, Switzerland

**Keywords:** COVID-19, SARS-CoV-2, Stillbirth, Late miscarriage, Classification, Placentitis

## Abstract

**Background:**

Stillbirth has been recognized as a possible complication of a SARS-CoV-2 infection during pregnancy, probably due to destructive placental lesions (SARS-CoV-2 placentitis). The aim of this work is to analyse stillbirth and late miscarriage cases in unvaccinated pregnant women infected with SARS-CoV-2 during the first two waves (wild-type period) in Belgium.

**Methods:**

Stillbirths and late miscarriages in our prospective observational nationwide registry of SARS-CoV-2 infected pregnant women (*n* = 982) were classified by three authors using a modified WHO-UMC classification system for standardized case causality assessment.

**Results:**

Our cohort included 982 hospitalised pregnant women infected with SARS-CoV-2, with 23 fetal demises (10 late miscarriages from 12 to 22 weeks of gestational age and 13 stillbirths). The stillbirth rate was 9.5‰ for singleton pregnancies and 83.3‰ for multiple pregnancies, which seems higher than for the background population (respectively 5.6‰ and 13.8‰). The agreement between assessors about the causal relationship with SARS-Cov-2 infection was fair (global weighted kappa value of 0.66). Among these demises, 17.4% (4/23) were “certainly” attributable to SARS-CoV-2 infection, 13.0% (3/23) “probably” and 30.4% (7/23) “possibly”. Better agreement in the rating was noticed when pathological examination of the placenta and identification of the virus were available, underlining the importance of a thorough investigation in case of intra-uterine fetal demise.

**Conclusions:**

SARS-CoV-2 causality assessment of late miscarriage and stillbirth cases in our Belgian nationwide case series has shown that half of the fetal losses could be attributable to SARS-CoV-2. We must consider in future epidemic emergencies to rigorously investigate cases of intra-uterine fetal demise and to store placental tissue and other material for future analyses.

**Supplementary Information:**

The online version contains supplementary material available at 10.1186/s12884-023-05624-3.

## Background

In March 2020, the World Health Organisation (WHO) declared COVID-19, the disease caused by the severe acute respiratory syndrome coronavirus-2 (SARS-CoV-2), a pandemic. Initially, there was little knowledge about the impact of a SARS-CoV-2 infection on pregnancy. Numerous questions arose about clinical management and expected outcomes [1, 2] as severe adverse perinatal consequences had been observed with other coronaviruses like SARS and Middle East respiratory syndrome (MERS) [1–3].

Accumulating evidence now shows that pregnant women must be considered a vulnerable group in case of a SARS-CoV-2 infection [4–12]. They have an increased risk of intensive care admission, ventilatory support need, and death compared with age-matched non-pregnant women [4–6, 12, 13]. Severe COVID-19 seems to be associated with iatrogenic preterm delivery, predominantly for maternal indication, and more neonatal admissions [4, 5, 7, 9, 10, 12]. Perinatal and transplacental transmission has been described but remains rare and is mostly associated with good neonatal outcomes [7, 14].

At first, stillbirth was not recognized as a complication of COVID-19 disease during pregnancy [11, 15]. Furthermore, some authors attributed the surge of stillbirths to an indirect impact of the pandemic (including restricted access to care and fewer prenatal visits) [16, 17], while others described a reduction in stillbirths in the whole population [18, 19]. There is now mounting evidence that SARS-CoV-2 infection and adverse fetal events are associated [15, 20–22]. In November 2021, the U.S. Center for Disease Control and Prevention warned about an increased risk of stillbirth with COVID-19, especially with the B.1.617.2 (Delta) variant compared to pregnant women without COVID-19 infection [21]. This increased risk of intra-uterine fetal demise (IUFD) exists regardless of the severity of symptoms in pregnant women and seems highest in the two to four weeks after infection [22, 23]. Several reports have described severe placental lesions, possibly explaining stillbirth due to SARS-CoV-2 infection [15]. The introduced term, SARS-CoV-2 placentitis, refers to the following triad of findings: chronic histiocytic intervillositis (CHI), perivillous fibrin deposition (sometimes massive) (MPFD), and villous trophoblast necrosis (TN) [15, 24–26].

The definition of stillbirth varies worldwide (gestational age cut-off, exclusion or not of late terminations of pregnancies (TOPs), …) [27–29]. Consequently, there is a high degree of heterogeneity in the stillbirth rates across countries, even between countries with similar socio-economic status, living conditions, and healthcare systems [28–30]. Early stillbirths are more likely to be missing from statistical reports [29].

Some risk factors for stillbirth (ethnicity, maternal obesity, hypertension, …) [30, 31] are the same risk factors as for severe SARS-CoV-2 infection during pregnancy [4, 11]. The pathologic examination of the placenta seems to be crucial to enable us to determine the likely cause of the demise [15, 32–34].

During the COVID-19 pandemic, we aimed to inform patients about the risks associated with a SARS-CoV-2 infection during pregnancy, and this information was lacking at the beginning of the pandemic.

The objective of this study was to evaluate whether SARS-CoV-2 infection is causing stillbirth and late miscarriages.

## Materials and methods

We conducted a nationwide population-based prospective observational register between the 1^st^ of March 2020 and the 28^th^ of February 2021, within the Belgian Obstetrical Surveillance System (B.OSS). Since 2012, B.OSS registers and analyses rare disorders and complications of pregnancy in Belgium, with the participation of all but one of the 102 Belgian maternity units [35], covering 97.4% of births. Each maternity unit has appointed a professional who is responsible for the recording of cases. A monthly reminder is sent by email to the network of these B.OSS collaborators. At the beginning of the pandemic, the surveillance system was activated to collect information on pregnant women infected with SARS-CoV-2.

During the peak incidence of the pandemic, weekly email reminders were sent. The appointed person in each maternity was asked to notify whether there had been a case or not. Any woman, either pregnant or in the first 42 days of her postpartum period, diagnosed with a SARS-CoV-2 infection, who was admitted to the hospital, was included. We considered only maternal infections confirmed by the detection of viral RNA by polymerase chain reaction (PCR) testing on a (naso)pharyngeal swab or aspirate in the last six weeks before or during hospitalization. Hospitalization could be for any cause including SARS-CoV2-infection, pregnancy-related complications or delivery. A total of 201 cases were excluded for the following reasons: no data collection form (*n* = 101); more than six weeks delay between the diagnosis of the infection and their hospitalisation (*n* = 68); no confirmation of SARS-Cov-2 infection (*n* = 18); no hospitalisation (*n* = 8); duplicates (*n* = 3); registration outside of the study period (*n* = 3). Detailed information was recorded with an online data collection form, designed by the International Network of Obstetric Survey Systems (INOSS) [5]. For each case included, maternal characteristics, details of the SARS-CoV-2 infection and its management, maternal outcomes, pregnancy outcomes, and neonatal outcomes were recorded.

Country of origin was extracted from patient records. Body mass index calculation was based on the first recorded weight during pregnancy. Tested samples (amniotic fluid, cord blood, placenta, high vaginal swabs, faeces, or other) and autopsy results were recorded. In case of a stillbirth, the collaborator was contacted to obtain the report of the pathological examination of the placenta.

We used an internationally accepted definition of stillbirth which includes all fetal demises at gestational ages ≥ 22 weeks or with a birth weight ≥ 500 g [28, 29]. We decided to also include miscarriages from 12 weeks onwards to capture a picture as broad as possible of the possible impact of SARS-CoV2-infection on pregnancy.

We used a modified classification, based on the WHO-Uppsala Monitoring Centre (UMC) system for standardised case causality assessment for adverse drug reactions (ADR) [36], to determine whether a case of stillbirth could be caused by the SARS-CoV2-infection or not (Table 1).

**Table 1:** Classification of stillbirths due to SARS-CoV-2 infection, modified from the WHO-UMC system for standardized case causality assessment

Causality term	Assessment criteria
Certain	Event with **plausible** time relationship with COVID infection **Cannot be explained by other disease or other event** **Supporting laboratory evidence**
Probably / Likely	Event with **reasonable** time relationship with COVID infection **Unlikely** to be attributed to disease or **other** event
Possible	Event with **reasonable** time relationship with COVID infection **Could** also be explained by disease or other event
Unlikely	Event with a time relationship with COVID infection that make an association **improbable** (but not impossible) Disease or other event provide plausible explanations
Unrelated	Event is clearly **NOT** related to the COVID infection

Initially, all cases were evaluated by three obstetricians (evaluators), working in the high-risk obstetric department of University Hospitals. After classification, if there was non-agreement, cases were reviewed to reach a consensus among the three evaluators. Another group of three obstetricians (reviewers), also working in the high-risk obstetric department of University Hospitals, revised the consensus classification of cases, to verify their agreement with the evaluators.

The reliability of agreement between the three evaluators was assessed by calculating Fleiss' Kappa with the ReCal3 0.1 software (http://dfreelon.org/recal/recal3.php). Statistical analyses were performed using STATA version 17 (Statacorp, TX, USA). Descriptive analyses were performed with presentation of numbers and proportions. A narrative descriptive approach is used to summarize the cases.

The study was approved by the central Ethics Committee of the University Hospital of Ghent (Ref. Number B670201526875), and local Ethics Committees gave their approval to the central ethics committee.

## Results

### Characteristics of the nationwide population-wide cohort

Between the 1^st^ of March 2020 and the 28^th^ of February 2021, 982 registration forms were completed for cases of hospitalised women with SARS-CoV-2 infection confirmed by PCR in pregnancy or the post-partum period in Belgium. Among these cases, 92 of the pregnant women (9.4%) were hospitalized for a severe SARS-CoV-2 infection, the remaining for an obstetrical reason (delivery or complication during pregnancy or shortly after). Two-thirds (655/982; 66.7%) were asymptomatic for SARS-CoV-2. The rate of first-trimester miscarriage was 0.5% (5/982). Ten women had a late miscarriage (from 12 to 22 weeks; 10/982; 1.0%). Twenty (20/982; 2.0%) of them were pregnant with twins. After 22 weeks of gestational age (GA), thirteen in-utero fetal demises were recorded (13/982; 1.3%).

The mean maternal age of women who had a late miscarriage or IUFD was 32.4 years old (standard deviation—sd 6.0). A total of 31.8% of pregnant women suffering from a stillbirth were 35 years or older. A minority of them were born in Belgium (40.9%) although 81.8% had Belgian citizenship. The median body mass index (BMI) at booking was 25; 31.6% of them were obese (BMI > 30) and 26.3% were overweight (BMI between 25 and 30).

The latest data available on the stillbirth rate in Belgium date from 2020, the year of the inclusion of the majority of the participants in our study. Stillbirth rates were 5.6/1000 and 13.8/1000 deliveries for singletons and multiple gestations, respectively (these rates include infants born after 22 weeks or with a birthweight of >  = 500 g and TOPs) [37–39]. In our cohort, for singletons, we recorded nine stillbirths out of 951 infected pregnancies (9.5‰). For multiple pregnancies, we observed three stillbirths out of a total of 36 born infants (83.3‰).

### Characteristics of late miscarriage and stillbirth cases

We observed ten cases of late miscarriage (10/962 – 10.4‰) and nine cases of IUFD (9/951 – 9.5‰) in singleton pregnancies. The details of the cases are listed in Supplementary Tables S1 to S4. In three of the 19 cases, the pregnancy was interrupted for fetal malformation (one case before 22 weeks). After the exclusion of TOP cases, a total of 16 cases were further analysed. In the non-TOP cases, one woman was diagnosed with gestational diabetes, and two others with preeclampsia. All but one woman delivered vaginally. In this case (case 5) the woman was admitted with progressive breathlessness at 37w3d. An urgent C-section was indicated for respiratory distress, despite the diagnosis of stillbirth. She was hospitalized in the intensive care unit for severe COVID infection for four days postpartum.

In ten cases (10/16), the placenta was sent for pathological examination. In five of these ten cases, findings suggestive of SARS-CoV-2 placentitis (CHI, MPFD, TN) were described (Table 2). In one case, acute funisitis and deciduitis were described, which could be related to the SARS-CoV-2 infection, but no other analyses were performed to confirm this hypothesis.

**Table 2:** Placental findings: case number, presence or absence of placental findings suggestive of SARS-CoV-2 are listed alongside the microbiology and immunohistochemistry results

Case	Histopathological examination				Microbiology and IHC
	**CHI**	**MPFD** **or FD**	**TN**	**Other findings**	
# 1	Yes	Yes	No	/	SARS-CoV-2 PCR neg Culture negative
# 2	No	Yes	Yes	MVM Placental infarcts Chorionitis	Vaginal SARS-CoV-2 PCR neg
# 3	No	No	No	Acute funisitis Acute deciduitis	NA
# 4	Yes	Yes	No	IHC: CD3 + T-lymphocytes CD68 + histiocytes	NA
# 5	No	Yes	No	Placental infarcts	NA
# 8	Yes	No	No	Placental atrophy Focal infarct zone	SARS-CoV-2 PCR neg
# Twin1	Yes	Yes	Yes	MVM 70–80% infarction	IHC positive SARS-CoV-2 nucleocapsid

*Abbreviations*: *CHI* Chronic histiocytic intervillositis, *MPFD* Massive perivillous fibrin deposition, *FD* Fibrin deposition, *TN* Trophoblast necrosis, *MVM* Maternal vascular malperfusion, *NA* Non applicable, *PCR* Polymerase chain reaction, *IHC* Immunohistochemistry, *CD* Cluster of differentiation, *neg* negative

### Characteristics of stillbirth cases in twin pregnancies

The characteristics of the stillbirth cases in twins are described in Table S5. In all cases obstetrical risk factors for stillbirth, other than SARS-CoV-2, were present.

There were four stillbirths and one death from perinatal asphyxia probably related to respiratory distress and SARS-CoV-2 infection. In this particular case, a C-section was performed for an abnormal fetal cardiotocogram of the twin that was alive on admission. The placenta was analysed and showed chronic histiocytic intervillositis, massive perivillous fibrin deposition, trophoblast necrosis and maternal vascular malperfusion. Immunohistochemistry (IHC) was positive for SARS-CoV-2 infection (positive for the nucleocapsid). The second case of twins had a positive PCR test on the placenta.

### Classification

Three obstetricians independently classified all cases according to the above-mentioned classification. We were unable to classify case “twin 3”, as essential information was missing (chorionicity, birth weight).

Agreement was evaluated with a global weighted kappa value of 0.66. The 2 × 2 kappa values between the three evaluators were 0.74 – 0.62 – 0.62. Afterwards, the consensus classification was reviewed by three other obstetricians (Table 3).

**Table 3:** Consensus classification of the stillbirth cases with the agreement between the three evaluators before reaching consensus and the agreement between reviewers with the consensus

Case	Agreement between evaluators	Consensus	Agreement between reviewers	Explanation
# 1	1/3	Certain	2/3	Some considered cases with very suggestive placental findings as “certain”, others preferred probable as there was no identification of SARS-CoV-2 on the placenta
# 2	3/3	Certain	1/3	Two reviewers preferred: “probable” because of IUGR and no identification of SARS-CoV-2 on the placenta
Twins 1	3/3	Certain	3/3	
Twins 2	3/3	Certain	2/3	One reviewer classified “possible” as no placental examination had been performed and because of low birth weight in both infants
# 3	1/3	Probable	1/3	Woman with type 1 diabetes and keto-acidosis. Some considered that keto-acidosis might have been caused by COVID; others preferred “possible” due to presence of other risk factors for IUFD
# 4	1/3	Probable	2/3	Half preferred “possible”
# 5	2/3	Probable	1/3	Half preferred “possible”; preeclampsia could have caused the IUFD
# 6	1/3	Possible	3/3	PPROM and chorio-amnionitis can be caused by other pathogens (no identification was performed)
# 7	3/3	Possible	3/3	
# 8	2/3	Possible	2/3	Incoherent timing; several other RF
# 9	2/3	Possible	3/3	PPROM and chorio-amnionitis can be caused by other pathogens (no identification was performed)
# 10	3/3	Possible	2/3	One considered “unlikely” as placental examination was normal
# 11	2/3	Possible	2/3	RF: past history of several mid-trimester losses, cervical incompetence?
# 12	1/3	Possible	2/3	Two considered it “unlikely”, because of severe preeclampsia. One considered “probable” as preeclampsia could have been associated with COVID
# 13	3/3	Unlikely	2/3	A reviewer preferred “possible” as no pathological examination of placenta was performed and there was no other explanation for IUFD
# 14	3/3	Unlikely	3/3	
# 15	3/3	Unlikely	3/3	
# 16	2/3	Unlikely	2/3	Incomplete investigation, so according to 2/6 a COVID infection could not be excluded (“possible”)

TOP cases were excluded (all evaluators and reviewers agreed on “unrelated”)

*Abbreviations*: *RF* Risk factor, *IUGR* Intra-uterine growth restriction, *IUFD* Intra-uterine fetal demise, *PPROM* Premature preterm rupture of membranes, *PCR* Polymerase chain reaction

Three cases were categorized as “unrelated”, all these cases were TOPs for congenital malformations (100% agreement).

For 22% of cases (5/23 fetuses), the SARS-CoV-2 infection was “certainly” the cause of the stillbirth. Three (14%) and seven pregnancies (32%) were classified in the probable and possible categories, respectively. According to our assessment, in nearly two thirds of cases (65% of the fetuses) evidence was available to suspect SARS-CoV-2 infection as the cause of the fetal demise. In all certain / probable cases, the gestational age was above 22 weeks. In the category “possible” all demises occurred before 22 weeks except case 12, with the IUFD occurring at 33 weeks’ gestation.

Four cases were classified as “unlikely”. In these cases, there was a reasonable other explanation for the stillbirth. The evaluators agreed almost completely for these cases, only one obstetrician would have classified case 16 as “possible” (Table 3). After discussion, the evaluators agreed to classify the case as “unlikely” because of the presence of multiple risk factors for stillbirth (advanced maternal age, multiparity, overweight, 40 weeks’ gestation). As there was no additional information (no autopsy, no pathological examination of placenta), one reviewer preferred to classify the case also as being “possible”. One reviewer of the consensus classification preferred to class case 13 as “possible”, instead of “unlikely”, as no placental investigation had been performed and no other explanation was present for the IUFD.

Four cases with supportive laboratory evidence were classified as “certain”: there was a 100% agreement between evaluators for three out of four cases; case 1 was also, after discussion, classified as “certain”. Two reviewers also classified case 1 as “probable”: the very suggestive placental findings were considered as “certain” for some; others, because of no identification of the virus on the placental side, classified the case as “probable”.

The two sets of twins (1 and 2) had a compatible history and SARS-CoV-2 was detected on the placenta (twins 1 by nucleocapsid identification by IHC, twins 2 by PCR). There was complete agreement between evaluators for these two cases as “certainly” attributable to COVID. One reviewer preferred to use “probable” for case “Twins 2”.

All the other cases were classified as “probable” or “possible”. In some cases, as several risk factors were present, the semantic difference between probable (unlikely other cause) or possible (could be another cause) was small and so an agreement was not obtained.

## Discussion

The risk of late miscarriage or stillbirth was increased in SARS-CoV-2 infected women, compared to the general population during the same period. Some of the IUFD can be attributed to the SARS-CoV-2 infection, but sometimes it was difficult to assess the causal linkage, especially when the placental examination was missing.

We observed a low rate of early miscarriages in our cohort. Most women who experience a first-trimester miscarriage will not be hospitalized, and thus not included in the registry, which underestimates the risk associated with the infection. A recent prospective cohort study that analysed, by self-reporting, the rate of miscarriage in pregnant women showed a, not statistically significant, higher risk in the infection group, compared to non-infected women (relative risk 1.7, 95% CI 1.0–3.0) [40].

Stillbirth rates in Belgium in 2020 have not changed compared to pre-pandemic rates (2011–2019) [37–39]. In this cohort of women with SARS-CoV-2 infection, we observed higher rates of stillbirth than those observed for singletons and multiple pregnancies in Belgium [29, 41]. In our cohort, as pregnant women have more risk factors (including age, BMI, and comorbidities), they are more susceptible to a severe SARS-CoV-2 infection but are also more likely to have a stillbirth [4, 5, 7, 11]. As numbers are small, it is not feasible to perform multivariate analysis for confounding factors.

Globally, more than 81 classification systems exist to determine the cause of stillbirths and neonatal deaths, 27 of them are more widely used [27]. There is still a need for a universal and straightforward classification system [27, 42]. The WHO has developed the International Classification of Diseases ICD-perinatal mortality classification which is promising. It classifies perinatal deaths with the possibility of a link to maternal conditions [27, 43]. Unfortunately, this classification was not suitable for our study, as a clear link to SARS-CoV-2 infection was not possible. The WHO-UMC case causality assessment for ADR is an internationally accepted classification that helps to determine the likelihood of a relationship between an adverse reaction and a drug. During the last two years, evidence has been gathered on the impact of SARS-CoV-2 infection on pregnancy. More and more a link between the infection and stillbirth cases is considered possible. With the modified WHO-UMC classification tool, more than 1/5 of cases were classified as “certainly” caused and more than half of the cases as “probably” or “possibly” caused by the SARS-CoV-2 infection. The good agreement between reviewers suggests a strong probability of this causal relationship.

SARS-CoV-2 placentitis is a triad of CHI, MPFD, and TN. This excessive inflammation leads to vascular injury and impaired perfusion which can cause chronic hypoxia in the fetus [15, 24, 25, 44, 45]. Given there is no treatment available to prevent placental damages caused by the virus, personal protection and vaccination remain important [8]. Favre et al. encouraged the monitoring of fetal growth and uteroplacental and fetal Dopplers in pregnant women who recovered from a COVID-19 infection [46]. Early guidelines of the International Society of Ultrasound in Obstetrics and Gynaecology advised evaluating fetal growth and amniotic fluid with umbilical artery Doppler if necessary [47]. Anuk et al. showed in their cohort increased pulsatility and resistance indices in the uterine and umbilical arteries in recovered COVID-19 patients compared to controls, revealing a possible effect on fetoplacental circulation [48]. Prepandemic studies documented the role of uteroplacental and fetal Doppler analyses in the prediction of late-onset fetal growth retardation and stillbirth [49, 50]. Uteroplacental and fetal Dopplers studies are the cornerstone of the monitoring of pregnancies in case of COVID-19 infection.

A limitation of the study is that a detailed investigation of the cause of death was not performed for all cases. Laboratory and pathology investigations are crucial in the search for the cause of a stillbirth or a late miscarriage [32]. Maternal infection (TORCH – acronym for Toxoplasmosis, Other, Rubella, Cytomegalovirus, Herpes) can cause stillbirth. More recently Ebola and Zika viruses were added to the list of pathogens that can harm the fetus [15, 51, 52]. In these infections, it is the pathogen (virus or parasite) that crosses the placental barrier and infects the fetal organs [15, 24]. Recent research shows that, in case of SARS-CoV-2 IUFD, there is an infection of the placenta that leads to its destruction [24, 25, 45]. In cases classified as “certain” or “probable”, we note that all, but one, had a pathologic investigation of the placenta with features of SARS-CoV-2 placentitis. The latter case (twin 2) had a SARS-Co-2 positive PCR test on the placenta. The placental examination was missing in four “possible” cases or abnormalities were not considered significant (two cases). If a pathological examination of the placenta was performed, some cases could have been classified differently; this underlines the need for a thorough investigation to determine the cause in case of a stillbirth [32]. Some cases lacked rigorous investigation, making classification and thus causality difficult to analyse. It would be important for future emerging infectious diseases to store placental and fetal material to be analysed later when diagnostic tools are more sophisticated. Another limitation of our study is that our cohort only analyses the impact of the wild-type variant of SARS-CoV2 and not of the other variants/ mutations [15, 21, 24].

At the beginning of the pandemic in Belgium, there was a shortage of testing materials. Consequently, only symptomatic patients were tested. Later in the pandemic, each hospital had its testing strategy (universal for hospitalisation or only when symptomatic) and this also changed over time. It is possible that some cases were not recorded, especially at the beginning of the pandemic. Another possible limitation of our study is that cases were excluded when the positive PCR dated from more than six weeks before hospitalisation. Late consequences of SARS-CoV-2 infection earlier in pregnancy could therefore be underestimated.

The strength of our study is that recorded cases of late miscarriages and stillbirths were collected nationwide during the wild-type period and in unvaccinated pregnant women, which allows us to evaluate the impact of SARS-CoV-2 infection during pregnancy.

## Conclusions

In women with SARS-CoV-2 infection, late miscarriage and stillbirth were attributable to the virus in half of the cases. When pathological examination or identification of the virus on the placenta was available, the link was unequivocal, which underlines the importance of thorough investigations in case of intra-uterine fetal demise.

## Supplementary Information

**Additional file 1:** **Table S1.** Characteristics of cases with intra-uterine fetal demise from pregnant women with SARS-CoV-2 infection (Cases #1-5). **Table S2.** Characteristics of cases with intra-uterine fetal demise from pregnant women with SARS-CoV-2 infection (Cases #6-10). **Table S3.** Characteristics of cases with intra-uterine fetal demise from pregnant women with SARS-CoV-2 infection (Cases #11-15). **Table S4.** Characteristics of cases with intra-uterine fetal demise from pregnant women with SARS-CoV-2 infection (Cases #16-19). **Table S5.** Characteristics of cases with at least one intra-uterine fetal demise from pregnant women with SARS-CoV-2 infection expecting twins (Cases #TWIN1-TWIN3).

## Data Availability

Data cannot be shared publicly because of confidentiality issues and potential identifiability of sensitive data as identified within the Research Ethics Committee application / approval. Requests to access the data can be made by contacting Karolien.benoit@b-oss.be.

## Abbreviations

COVID-19: Coronavirus Disease 19
WHO: World Health Organisation
IUFD: Intra-uterine fetal demise
SARS-COV-2: Severe Acute Respiratory Syndrome coronavirus-2
MERS: Middle East Respiratory Syndrome
U.S.: United States of America
CHI: Chronic histiocytic intervillositis
TOPs: Terminations of pregnancy
B.OSS: Belgian Obstetrical Surveillance System
RNA: Ribonucleic acid
PCR: Polymerase chain reaction
INOSS: International Obstetrical Surveillance System
WHO-UMC: World Health Organisation Uppsala Monitoring Centre
ADR: Adverse drug reactions
GA: Gestational age
sd: Standard deviation
BMI: Body mass index
TN: Trophoblast necrosis
IHC: Immunohistochemistry
RF: Risk factor
IUGR: Intra-uterine growth restriction
PPROM: Premature preterm rupture of membranes
ICD: International Classification of Diseases
TORCH: Acronym for toxoplasmosis, other, rubella, cytomegalovirus, herpes
