# Supplementary material for: Late miscarriage and stillbirth in asymptomatic and symptomatic hospitalised pregnant women in Belgium during the first and second waves of COVID-19: a prospective nationwide population-based study
Source: BMC Pregnancy Childbirth. 2023 May 16;23:356. doi: 10.1186/s12884-023-05624-3 (PMC10185936; doi:10.1186/s12884-023-05624-3)
Supplement: Supplementary file 1 — Additional file 1: Table S1. Characteristics of cases with intra-uterine fetal demise from pregnant women with SARS-CoV-2 infection (Cases #1-5). Table S2. Characteristics of cases with intra-uterine fetal demise from pregnant women with SARS-CoV-2 infection (Cases #6-10). Table S3. Characteristics of cases with intra-uterine fetal demise from pregnant women with SARS-CoV-2 infection (Cases #11-15). Table S4. Characteristics of cases with intra-uterine fetal demise from pregnant women with SARS-CoV-2 infection (Cases #16-19). Table S5. Characteristics of cases with at least one intra-uterine fetal demise from pregnant women with SARS-CoV-2 infection expecting twins (Cases #TWIN1-TWIN3). [file 12884_2023_5624_MOESM1_ESM.docx]

Supplementary tables

| **Characteristics** | **Case 1** | **Case 2** | **Case 3** | **Case 4** | **Case 5** |
| --- | --- | --- | --- | --- | --- |
| Maternal age | 24 yrs | 29 yrs | 34 yrs | 30 yrs | 27 yrs |
| Gestational age | 24w4d | 35w0d | 15w6d | 25w2d | 37w3d |
| Risk factors | Nullipara  Gastric bypass | / | BMI 44  Keto-acidosis during this pregnancy  Acute renal failure during this pregnancy  Gestational diabetes | / | BMI 40  Poor follow-up  Pregnancy induced hypertension 🡪 preeclampsia |
| Maternal COVID infection | 23w5d  high fever; anosmia;  sore throat | 31w5d fever , headache, flu, lethargy  32w5d more dyspnoea | 12w6d  vomiting | Asymptomatic | 36w3d  cough, sore throat, dyspnea |
| Maternal PCR for SARS-CoV-2 | Positive  23w5d | Positive  31w5d  Negative  35w0d | Positive  13w1d | Positive  25w2d | Positive  37w3d |
| Reason for hospital admission | Stillbirth | Stillbirth | Hyperemesis gravidarum &  Stillbirth | Stillbirth | Progressive breathlessness at 37w3d |
| Placental findings | CHI  MPFD | MPFD  TN; MVM  Infarct zones  Chorionitis | Acute funisitis  Acute deciduitis | FD  CHI | MPFD  Zones of infarct |
| Autopsy pathology findings | / | / | NSA  Fetus 15-16 wk | NSA | / |
| Placental status for SARS-CoV-2 | Negative | / | / | IHC: CD3+  T-lymphocytes; CD68+ histiocytes |  |
| Other analysis | Thrombocyto-penia  Placental culture negative | Vaginal PCR negative for SARS-CoV-2 | / |  |  |
| Delivery | vaginal | vaginal | vaginal | vaginal | C-section* |
| Birth weight | 550g | 1570g | 43g | 640g | 2345g |
| Consensus classification | Certain | Certain | Probable | Probable | Probable |

**Table S1.** Characteristics of cases with intra-uterine fetal demise from pregnant women with SARS-CoV-2 infection (Cases #1-5)

* Urgent pre-labour C-section for respiratory distress. Detection of stillbirth on admission. Four days care at intensive care unit.

Abbreviations: BMI= body mass index; CD= cluster of differentiation; CHI= chronic histiocytic intervillositis; C-section= caesarean section; FD= fibrin deposition; IHC= immunohistochemistry; MPFD= massive perivillous fibrin deposition; MVM= maternal vascular malperfusion; NSA= not-significant abnormalities; PCR= reverse transcription polymerase chain reaction; TN= trophoblast necrosis;

| **Characteristics** | **Case 6** | **Case 7** | **Case 8** | **Case 9** | **Case 10** |
| --- | --- | --- | --- | --- | --- |
| Maternal age | 33 yrs | 42 yrs | 40 yrs | 29 yrs | 37 yrs |
| Gestational age | 13w0d | 17w2d | 17w5d | 19w2d | 19w5d |
| Risk factors | / | No Belgian citizen  Maternal age | Country of birth not Belgium  Maternal age  BOH one late and one early MC | Nullipara  BMI 29  Origin Curaçao  2 early MC | Grande multipara (P4)  BMI 37,2  Sleeve gastrectomy  Mediterrean origin |
| Maternal COVID infection | 12w fever | 16w0d fever, cough, anosmia | Asymptomatic | Asymptomatic | 8w fever 10 days; headache; lethargy; anosmia |
| Maternal PCR for SARS-CoV-2 | Positive  12w0d | Positive  16w0d  Negative  16w6d | Positive  17w5d | Positive  19w2d | Positive  18w5d  NSA |
| Reason for hospital admission | PPROM  Chorioamnionitis | Pain | Stillbirth | PPROM | Stillbirth |
| Placental findings | Not performed | Refused | CHI  Placental atrophy Focal infarct zone | Not performed | NSA |
| Autopsy pathology findings | / | Refused | / | / | NSA |
| Placental status for SARS-CoV-2 | / | / | Negative | / | Negative (PCR) |
| Other analysis | / | / | CGH normal  AF culture negative | / | CGH normal  Vaginal culture negative  Placental culture negative |
| Delivery | vaginal | vaginal | vaginal | vaginal | vaginal |
| Birth weight | / | ? | 90g | / | / |
| Consensus classification | Possible | Possible | Possible | Possible | Possible |

**Table S2.** Characteristics of cases with intra-uterine fetal demise from pregnant women with SARS-CoV-2 infection (Cases #6-10)

Abbreviations: BMI= body mass index; BOH= bad obstetrical history; CGH= comparative genomic hybridization; CHI= chronic histiocytic intervillositis; MC= miscarriage; NSA= non-significant abnormalities; P= parity; PCR= polymerase chain reaction; PPROM= preterm premature rupture of membranes;

| **Characteristics** | **Case 11** | **Case 12** | **Case 13** | **Case 14** | **Case 15** |
| --- | --- | --- | --- | --- | --- |
| Maternal age | 25 yrs | 29 yrs | 30 yrs | 42 yrs | 34 yrs |
| Gestational age | 20w2d | 33w4d | 16w1d | 20w0d* (14w0d) | 28w0d |
| Risk factors | Nullipara  -  Multiple mid trimester losses | Grande multipara (P3)  -  HIV  -  Severe preeclampsia | BMI 38  Origin: Suriname | Grande multipara (P5)  -  Origin Kenya | BMI 28.2  -  Gastric bypass  -  Severe anemia at 14w (Hb 5,8 g/dL - 2 PRC transfusion)  -  Multiple vitamin deficiencies during pregnancy |
| Maternal COVID infection | Asymptomatic | 33w4d fever; lethargy; sore throat | Asymptomatic | Asymptomatic | Asymptomatic |
| Maternal PCR for SARS-CoV-2 | Positive  13w1d | Positive  PP day 6° | Positive  16w2d | Positive  20w3d | Positive  28w0d |
| Reason for hospital admission | Cerclage (13w2d) | In labour | Metrorrhagia  PPROM | Stillbirth | Pain 🡪 gastro-intestinal invagination |
| Placental findings | Low weight. No other info. | Not performed | Not performed | NSA. Placental weight cfr 11 wk | NSA |
| Autopsy pathology findings | NSA  Fetus cfr 16 wk | / | NSA  Fetus cfr 16 w | / | / |
| Placental status for SARS-CoV-2 |  | / | / | / | / |
| Other analysis | / | / | PCR stillborn anus / mouth / body negative  PCR UC negative | Maternal IgG SARS-CoV-2 positive at 20w3d | / |
| Delivery | vaginal | vaginal | vaginal | vaginal | vaginal |
| Birth weight | / | 2080g | 128g | / | 1070g |
| Consensus classification | Possible | Possible | Unlikely | Unlikely | Unlikely |
|  |  |  |  |  |  |

**Table S3.** Characteristics of cases with intra-uterine fetal demise from pregnant women with SARS-CoV-2 infection (Cases #11-15)

° Although symptoms, only tested 6 days postpartum. Late testing due to shortage of testing material in beginning of SARS-CoV2 pandemic.

* Ultrasound: CRL cfr 14 weeks of gestation.

Abbreviations: AF= amniotic fluid; BMI= body mass index; HB= hemoglobin; HIV= human immunodeficiency virus; NSA= not-significant abnormalities; P= parity; PCR= reverse transcription polymerase chain reaction; PPROM= premature preterm rupture of membranes; PRC= packed red blood cells (in case of transfusion); UC= umbilical cord;

| **Characteristics** | **Case 16** | **Case 17** | **Case 18** | **Case 19** |
| --- | --- | --- | --- | --- |
| Maternal age | 45 yrs | 35 yrs | 29 yrs | 35 yrs |
| Gestational age | 40w0d | 19w1d | 27w6d | 29w3d |
| Risk factors | Maternal age -  Multiparous (P4)  -  BMI 25.2  -  Origin India | Nullipara  -  Diabetes type 2 | / | / |
| Maternal COVID infection | Asymptomatic | Post MC day 2 Flu-like  Lethargy | Asymptomatic | Asymptomatic |
| Maternal PCR for SARS-CoV-2 | Positive  40wk0d | Positive  post MC day | Positive  26w6d | Positive  29w3d |
| Reason for hospital admission | Stillbirth | TOP | TOP | TOP  CC dysmorphism |
| Placental findings | Not performed | Not performed | Not performed | Not performed |
| Autopsy pathology findings | Not performed | hydrops, facial dysmorphia, complex cardiopathy | / | / |
| Placental status for SARS-CoV-2 | / | / | / | / |
| Other analysis | / | / | / | CGH PPPR6A1 mutation |
| Delivery | vaginal | vaginal | vaginal | vaginal |
| Birth weight | 3090g | ? | 1120g | 1000g |
| Consensus classification | Unlikely | Unrelated | Unrelated | Unrelated |
|  |  |  |  |  |

**Table S4.** Characteristics of cases with intra-uterine fetal demise from pregnant women with SARS-CoV-2 infection (Cases #16-19)

Abbreviations:
BMI= body mass index; CC= corpus callosum; CGH= comparative genomic hybridization; MC= miscarriage; P= parity; TOP= termination of pregnancy;

| **Characteristics** | **Case TWIN 1** | **Case TWIN 2** | **Case TWIN 3** |
| --- | --- | --- | --- |
| Maternal age | 33 yrs | 31 yrs | 28 yrs |
| Gestational age | 36w6d | 24w2d | 36w1d |
| Twin type | DCDA | Unknown | Unknown |
| Risk factors | Nullipara  -  Late booking  -  Preeclampsia  -  Growth discordance | Gestational diabetes | BMI 33,6  -  Gestational diabetes  -  Gestational hypertension |
|  |  |  |  |
|  |  |  |  |
| Maternal COVID infection | 36wk: fever, cough,  diarrhea, dyspnea, anorexia | 22w3d: fever  & rhinorrhoea | Asymptomatic |
| Maternal PCR for SARS-CoV-2 | Positive  35w6d | Positive  22w3d | Positive  35w5d |
| Reason hospital admission | Stillbirth twin 1;  Abnormal fetal monitoring twin 2 | Fulminant in labor  Pathological fetal monitoring twin 2 | Stillbirth twin 2 |
| Placental findings | CHI  MPFD  TN  MVM  70-80% infarct zones | Not performed | Not performed |
|  |  |  |  |
| Autopsy pathology findings | / | / | / |
| Placental status for SARS-CoV-2 | IHC positive SARS-Cov2 nucleocapsid | RT-PCR positive | / |
| Other analysis | AF culture negative | / | / |
| Delivery | Urgent C-section for abnormal monitoring T2 | Vaginal | Primary C-section |
| Birth weight | 1785g  2480g | 345g  375g | 2718g  Not mentioned |
| Fetal / Infant outcome | IUFD | IUFD | Alive |
|  | Neonatal death day 5 (asphyxia) | IPFD | IUFD |

**Table S5.** Characteristics of cases with at least one intra-uterine fetal demise from pregnant women with SARS-CoV-2 infection expecting twins (Cases #TWIN1-TWIN3)

Abbreviations:

AF= amniotic fluid; BMI= body mass index; CHI= chronic histiocytic intervillositis; DCDA= dichorionic diamniotic twin; IHC= immunohistochemistry; IPFD= intra-partum fetal death; IUFD= intra-uterine fetal death; MPFD= massive perivillous fibrin deposition; MVM= maternal vascular malperfusion; PCR= polymerase chain reaction; TN=trophoblast necrosis;
